# Regulation of adipose tissue lipolysis by ghrelin is impaired with high-fat diet feeding and is not restored with exercise

**DOI:** 10.1080/21623945.2021.1945787

**Published:** 2021-07-05

**Authors:** Barbora Hucik, Andrew J. Lovell, Evan M. Hoecht, Daniel T. Cervone, David M. Mutch, David J. Dyck

**Affiliations:** Department of Human Health and Nutritional Sciences, University of Guelph, Guelph, Ontario, Canada

**Keywords:** Ghrelin, adipose, high-fat diet, lipolysis, exercise, ATOC

## Abstract

Ghrelin is released from the stomach as an anticipatory signal prior to a meal and decreases immediately after. Previous research has shown that both acylated (AG) and unacylated (UnAG) ghrelin blunt adrenoreceptor-stimulated lipolysis in rat white adipose tissue (WAT) *ex vivo*. We investigated whether acute or chronic consumption of a high fat diet (HFD) impaired the ability of ghrelin to regulate adipose tissue lipolysis, and if this impairment could be restored with exercise. After 5 days (5d) of a HFD, or 6 weeks (6 w) of a HFD (60% kcal from fat) with or without exercise training, inguinal and retroperitoneal WAT was collected from anesthetized rats for adipose tissue organ culture. Samples were treated with 1 μM CL 316,243 (CL; lipolytic control), 1 μM CL+150 ng/ml AG or 1 μM CL+150 ng/ml UnAG. Incubation media and tissue were collected after 2 hours. Colorometric assays were used to determine glycerol and free fatty acid (FFA) concentrations in media. Western blots were used to quantify the protein content of lipolytic enzymes and ghrelin receptors in both depots. CL stimulated lipolysis was evidenced by increases in glycerol (p < 0.0001) and FFA (p < 0.0001) concentrations in media compared to control. AG decreased CL-stimulated glycerol release in inguinal WAT from 5d LFD rats (p = 0.0097). Neither AG nor UnAG blunted lipolysis in adipose tissue from 5d or 6 w HFD-fed rats, and exercise did not restore ghrelin’s anti-lipolytic ability in 6 w HFD-fed rats. Overall, this study demonstrates that HFD consumption impairs ghrelin’s ability to regulate adipose tissue lipolysis.

## Introduction

Ghrelin is classically known as an appetite-regulating hormone [1] It is produced in the stomach and released as an anticipatory signal prior to a meal and decreases immediately after. Recent studies expanded upon the classical role of ghrelin to demonstrate its regulatory effects on peripheral tissue (e.g. white adipose tissue, muscle) carbohydrate and lipid metabolism [2] 3, 4, 5, 6, 7, 8] . *Ex vivo*, ghrelin has been shown to blunt β adrenoreceptor-stimulated lipolysis in adipocytes [9], and in rat subcutaneous and visceral white adipose tissue (WAT) via the suppression of hormone-sensitive lipase activity (HSL) [5], a major enzyme regulating triglyceride hydrolysis. Although the acylated isoform of ghrelin (AG) is typically considered to be metabolically active, we have shown previously that the unacylated isoform of ghrelin (UnAG) has a greater role in the regulation of CL-stimulated lipolysis in WAT [5]. Both AG and UnAG blunt adrenergic-stimulated lipolysis and fatty acid reesterification in cultured adipose tissue of chow-fed rats [5] and ghrelin’s anti-lipolytic effect has also been observed in muscle [6]. Thus, ghrelin may be decreasing lipolysis in preparation for the upcoming storage of fatty acids following a meal (by inhibiting HSL), while simultaneously maintaining circulating fatty acid levels (by inhibiting WAT reesterification) to spare blood glucose.

To date, the specific regulatory role of ghrelin in WAT metabolism following high-fat feeding has not been studied. In response to high-fat diet (HFD) consumption, many tissues show altered membrane structure and an impaired response to many hormones (insulin, leptin, adiponectin) involved in the regulation of substrate metabolism. Given ghrelin’s role in regulating WAT lipolysis, we hypothesized that a HFD could contribute to the development of WAT resistance to ghrelin. Such resistance could result in impaired control of lipolysis and increased circulating fatty acids prior to a meal. However, it is unknown whether the consumption of a HFD can alter the ability of WAT to respond to ghrelin. Previous work in our lab using rats has demonstrated that ghrelin no longer stimulates oxidation in muscle to protect that tissue from fatty acid-induced impairments in insulin signalling following chronic HFD consumption [4]. This strongly suggests that HFD may cause ghrelin resistance in muscle. Whether this is true in adipose tissue needs to be determined, and is the focus of the current study.

Therefore, the purpose of this study was to investigate the role of both ghrelin isoforms (AG and UnAG) in mediating WAT lipolysis and fatty acid reesterification following acute and chronic high-fat feeding using an adipose tissue organ culture (ATOC) model. In addition, we also implemented an exercise training condition, as chronic exercise training is known to prevent or reduce the resistance to numerous hormones induced by a HFD [10] 11]. Further, exercise training has been documented to improve the control of basal lipolysis and release of circulating free fatty acids in obese individuals [12]. We hypothesized that both acute (5 days, 5d) and chronic (6 weeks, 6 w) consumption of a HFD would blunt the response of WAT to ghrelin, resulting in impaired suppression of β adrenoreceptor-stimulated lipolysis. Furthermore, we hypothesized that WAT response to ghrelin would be restored in chronic HFD fed rats with the implementation of a 4-week exercise training protocol.

## Results

### Effect of 5d and 6 w HFD on body weight, adiposity and caloric intake

Body weight did not significantly differ between 5d LFD and HFD rats (Table 2), although caloric intake was significantly increased with the HFD (p < 0.0001; Table 2). A 5d HFD significantly increased iWAT (p = 0.0305) and RP (p = 0.0110) adipose depot weights (Table 2).

**Figure 1.**
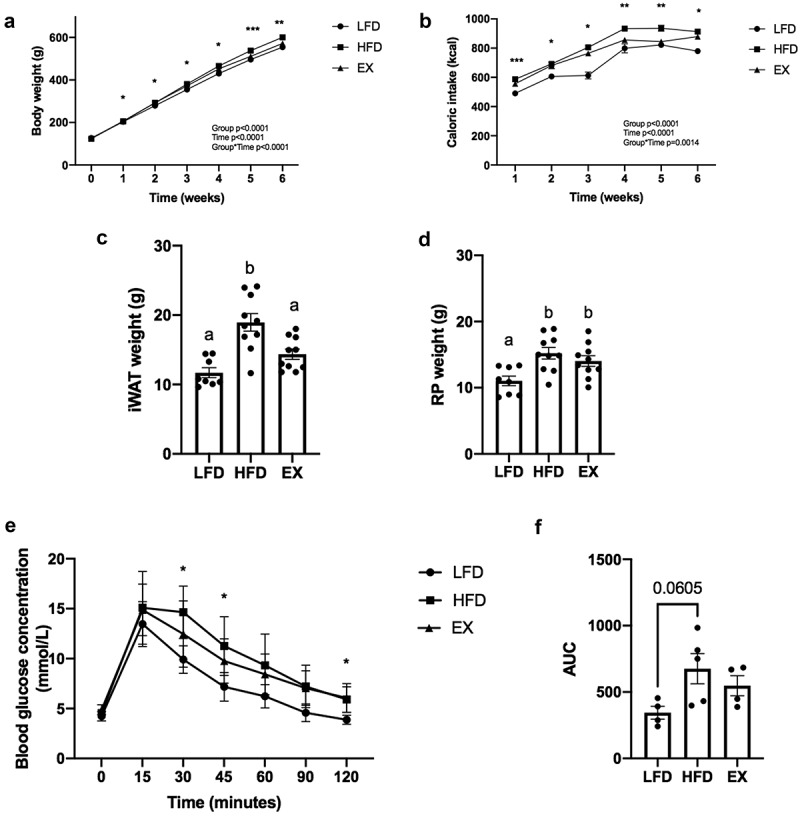
Body weight (Figure 1a), caloric intake (Figure 1b), iWAT and RP depot weights (Figure 1c-d) and blood glucose measurements (Figure 1e-f) in rats fed either a low-fat (LFD), or high-fat diet (HFD) with and without a 4-week exercise intervention. Bonferroni post hoc testing showed a significant increase in body weight in HFD-fed compared to LFD-fed rats. Asterisks (*) denote significant differences between treatment groups (p < 0.05). Data are presented as mean ± S.E.M. (n = 8–10 rats/group). Blood glucose was measured after an overnight (~8-10 h fast), before intraperitoneal injection of glucose and at 15, 30, 45, 60, 90 and 120 minutes post-injection. Bonferroni post hoc testing showed a significant difference in blood glucose between LFD and HFD at 30, 45 and 120 minutes post-injection, as indicated by asterisks. No significant difference was observed between EX and LFD or HFD groups. The AUC was analysed using an unpaired t-test. Data are presented as mean±S.E.M. (n = 4–6 rats/group)

Rats fed a 6 w HFD had significantly increased body weight compared to rats fed a 6 w LFD (p = 0.0075), but not 6 w HFD-fed rats undergoing a 4-week exercise training intervention (EX) (Figure 1a). Total caloric intake was significantly greater in the 6 w HFD group (p < 0.05) compared to both 6 w LFD and 6 w EX groups (Figure 1b). A 6 w HFD significantly increased (p < 0.05) iWAT and RP depot weights compared to 6 w LFD-fed rats (Figure 1c and 1d). A 4-week exercise training intervention resulted in decreased iWAT depot weight compared to rats fed a 6 w HFD (p = 0.0057; Figure 1c). To confirm a positive training response, muscle subunit IV of cytochrome c oxidase (COXIV) protein content was assessed. Trained rats (2.04 A.U. ± 0.29 S.E.M.) exhibited a 104% increase in COXIV relative to LFD-fed sedentary rats (1.0 ± 0.17; p = 0.006) and a 63% increase in COXIV relative to HFD-fed sedentary rats (1.25 ± 0.13; p = 0.04).

### Effect of 6 w HFD on whole-body glucose tolerance

6 w HFD-fed rats had significantly higher blood glucose levels compared to 6 w LFD-fed rats at 30 (p = 0.0152), 45 (p = 0.0485) and 120 (p = 0.0228) minutes (Figure 1e) during the intraperitoneal glucose tolerance test (IPGTT). There was a trend towards an increase in the area under the curve (AUC) during the IPGTT in rats fed a 6 w HFD compared to a 6 w LFD (p = 0.0605, figure 1f), demonstrating a decrease in glucose tolerance.

### Ghrelin blunts CL-induced lipolysis in iWAT in 5d LFD, but not HFD rats

**Figure 2.**
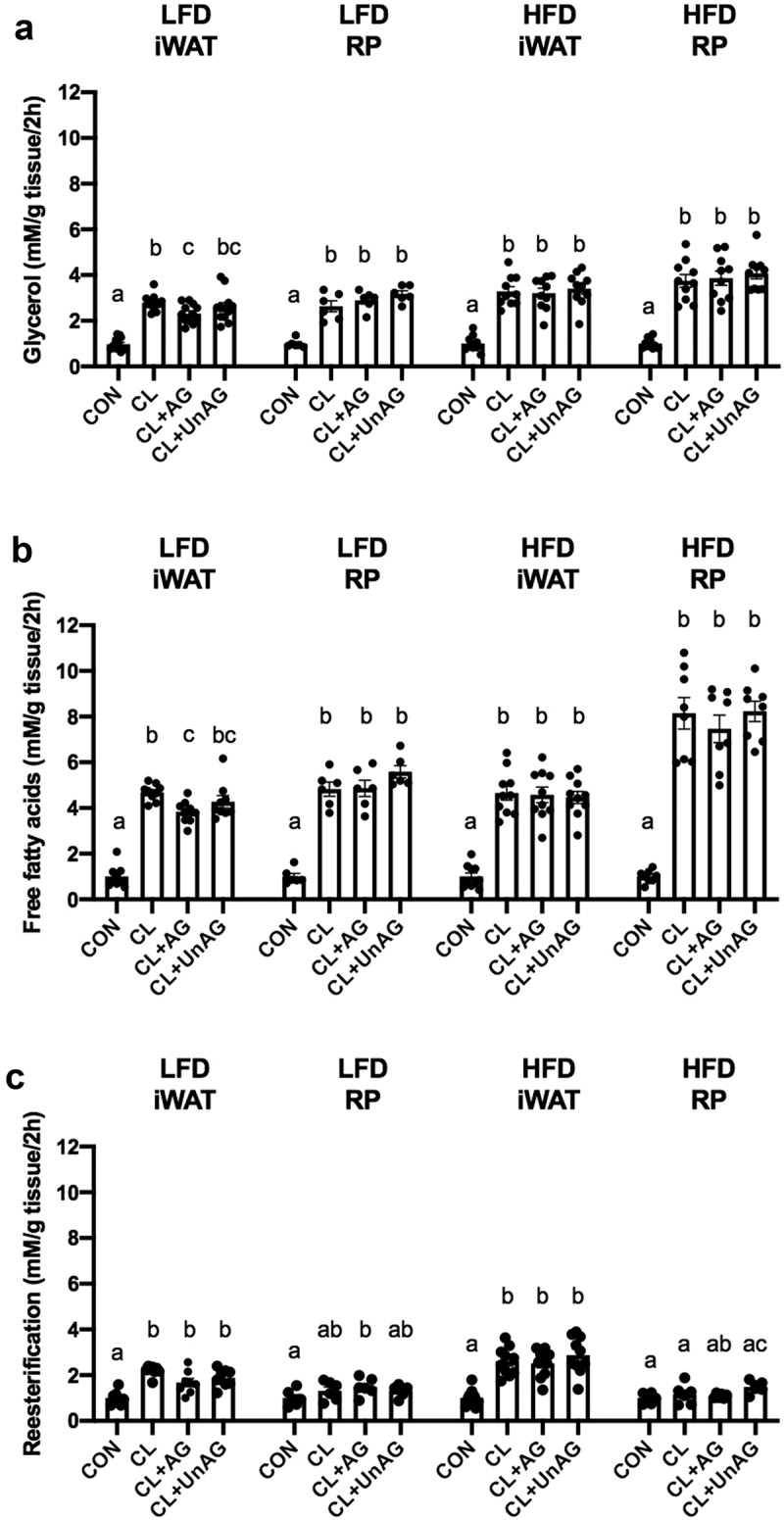
*Ex vivo* effects of CL, CL+AG and CL+UnAG compared to CON on glycerol release (Figure 2a), fatty acid release (Figure 2B) and reesterification (Figure 2 C) in both iWAT and RP after 5D of diet. Repeated measures one-way ANOVA showed significant differences in phosphorylation of HSL in treatments compared to CON. Superscript letters denote significant differences between treatment groups (p < 0.05). All data are presented as mean±S.E.M (n = 6–10 rats/group)

In iWAT from 5d LFD-fed rats, CL significantly increased glycerol (Figure 2a; p < 0.0001) and free fatty acid release (Figure 2b; p < 0.0001) compared to CON. AG decreased CL-stimulated glycerol (Figure 2a; p = 0.0097) and free fatty acid release (Figure 2b; p = 0.0111). In RP, CL significantly increased glycerol (Figure 2a; p = 0.0030) and free fatty acid release (Figure 2b; p = 0.0005) compared to CON, but neither AG nor UnAG decreased CL-stimulated glycerol or free fatty acid release (Figure 2a and 2b). In 5d HFD-fed rats, CL significantly increased glycerol (Figure 2a; p < 0.0001) and free fatty acid release (Figure 2b; p < 0.0001) in both iWAT and RP. However, neither AG nor UnAG were able to decrease CL-stimulated glycerol or free fatty acid release (Figure 2a and 2b).

In iWAT, CL significantly increased fatty acid reesterification in both 5d LFD-fed (p = 0.0009) and HFD-fed rats (p = 0.0003) compared to CON, but neither AG nor UnAG altered CL-stimulated fatty acid reesterification compared to CON (Figure 2c). In RP, there was no CL-stimulated increase in fatty acid reesterification in either 5d LFD-fed or HFD-fed rats (Figure 2c)

### Ghrelin does not blunt lipolysis in iWAT or RP AT from 6 w LFD and HFD rats

**Figure 3.**
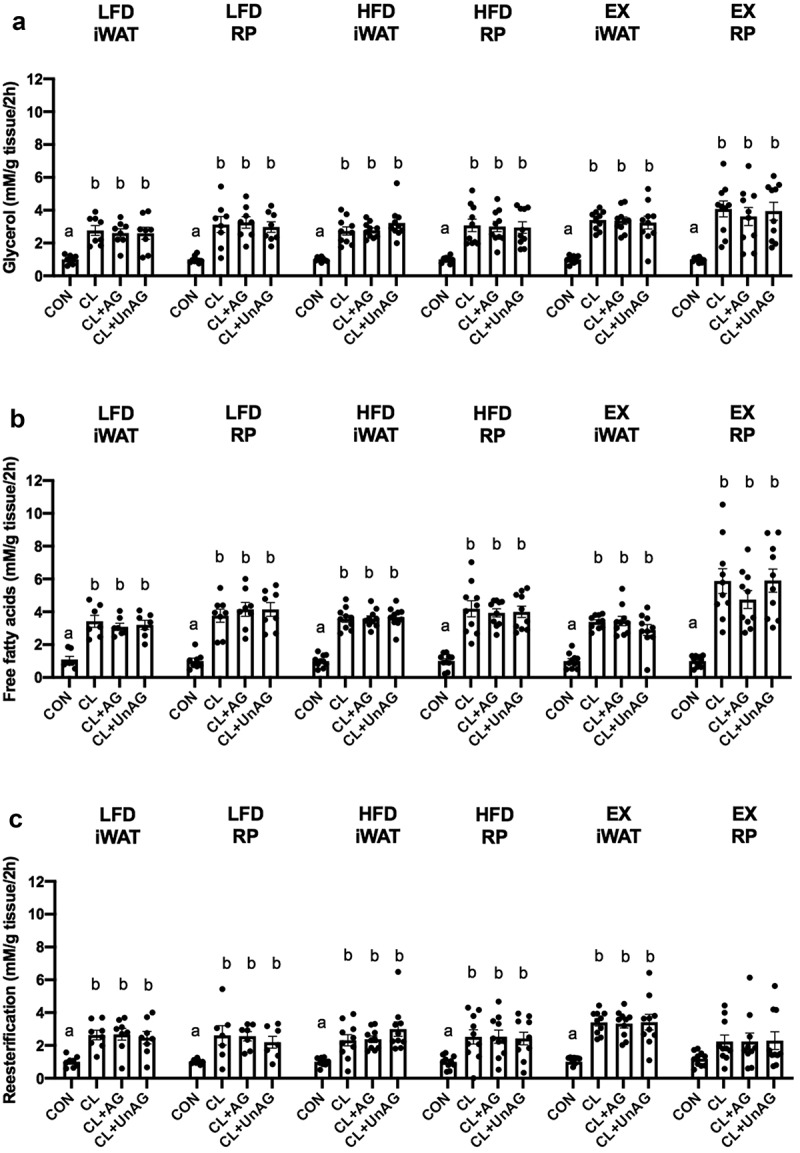
*Ex vivo* effects of CL, CL+AG and CL+UnAG compared to CON on glycerol release (Figure 3A), fatty acid release (Figure 3B) and reesterification (Figure 3 C) in both iWAT and RP after 6 weeks of diet. Repeated measures one-way ANOVA showed significant differences in phosphorylation of HSL in treatments compared to CON. Superscript letters denote significant differences between treatment groups (p < 0.05). All data are presented as mean±S.E.M (n = 7–10 rats/group)

In iWAT and RP from 6 w LFD-fed, HFD-fed and EX rats, CL significantly increased glycerol (Figure 3a; p < 0.05) and free fatty acid release (Figure 3b; p < 0.05) compared to CON. Both AG and UnAG failed to decrease CL-stimulated glycerol and free fatty acid release in all groups (Figure 3a and 3b).

In all 6 w experimental groups, CL significantly increased fatty acid reesterification (p < 0.0001) compared to CON, but neither AG nor UnAG blunted CL-stimulated fatty acid reesterification compared to CON (Figure 3c), with the exception of RP from 6 w EX rats, which did not show any significant CL-stimulated changes in reesterification (Figure 3c).

### Ghrelin does not blunt CL-stimulated HSL phosphorylation in iWAT or RP AT in 5d or 6 w experimental groups

**Figure 4.**
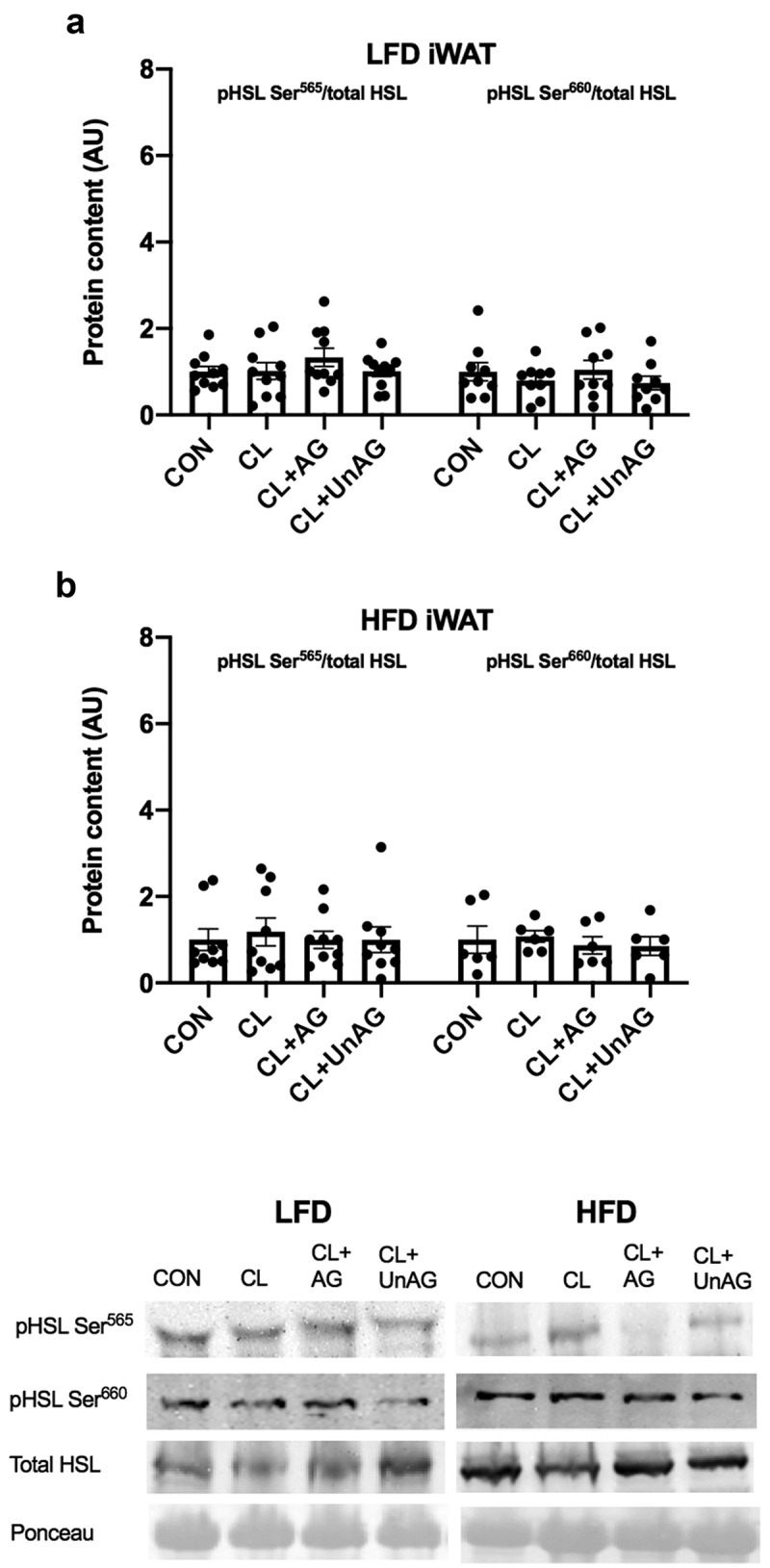
*Ex vivo* effects of CL, CL+AG and CL+UnAG compared to CON on phosphorylated HSL/total HSL in iWAT of 5d groups. Repeated measures one-way ANOVA did not show significant differences in phosphorylation of HSL in treatments compared to CON. Representative Western blots for all proteins are provided. All data are presented as mean arbitrary units (AU)±S.E.M (n = 8–9 rats/group)

**Figure 5.**
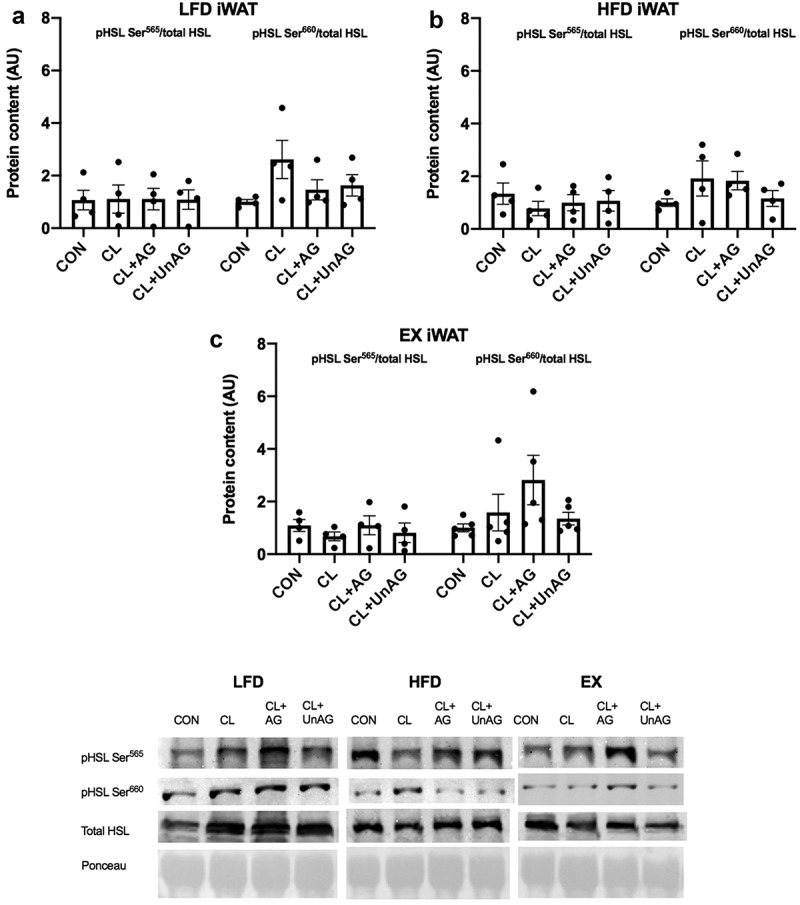
*Ex vivo* effects of CL, CL+AG and CL+UnAG compared to CON on phosphorylated HSL/total HSL in iWAT of 6 w groups. Repeated measures one-way ANOVA showed no significant differences in phosphorylation of HSL in treatments compared to CON. Representative Western blots for all proteins are provided. All data are presented as mean arbitrary units (AU)±S.E.M (n = 4–5 rats/group)

In iWAT and RP from 5d LFD-fed and HFD-fed rats, there were no differences with CL or CL+ghrelin treatments compared to the control group in the phosphorylation of HSL at activating residue Ser^660^ or inhibitory residue Ser^565^ (Figure 4a and 4b). After 6 w, there were no differences in the phosphorylation of HSL at the Ser^660^ or Ser^565^ residues in any treatments compared to the control group in either iWAT or RP depots from LFD-fed, HFD-fed and EX groups (Figure 5a and 5b).

### Ghrelin receptor expression in AT following HFD and exercise training

**Figure 6.**
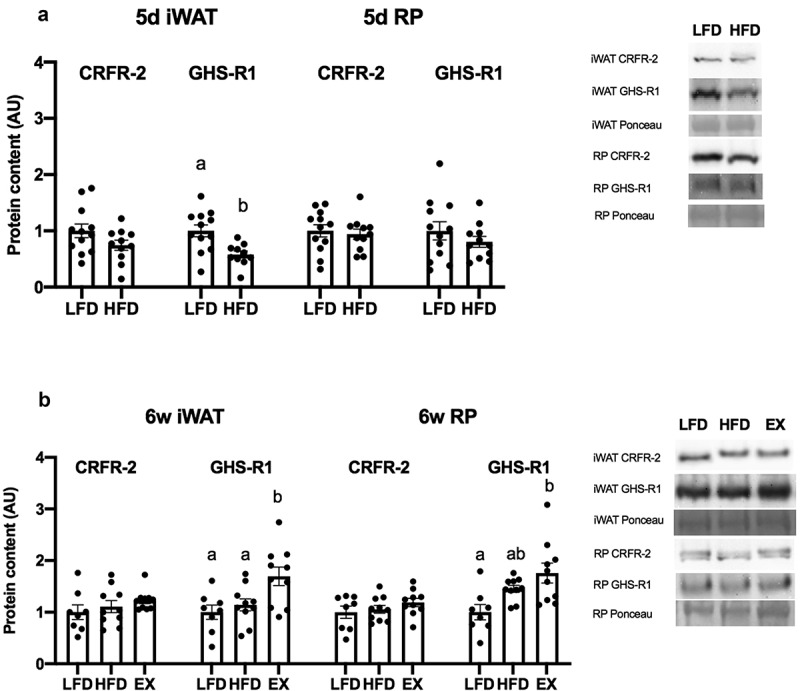
Protein content of ghrelin receptors CRFR-2 and GHS-R1 in iWAT of 5d groups (Figure 6A) and 6 w groups (Figure 6B). Protein content was analysed using an unpaired t-test. Superscript letters denote significant differences between treatment groups (p < 0.05). Representative Western blots for all proteins are provided. All data are presented as mean arbitrary units (AU)±S.E.M (n = 10–12 rats/group)

The main ghrelin receptor is GHS-R1 [13]; however, a previous report suggested that ghrelin may exert some effects in skeletal muscle through CRFR-2 [14]. Therefore, we examined the expression of both receptors in WAT. There were no significant differences in CRFR-2 expression in iWAT or RP from HFD-fed rats compared to LFD-fed after 5d (Figure 6a). Following 5d of a HFD, GHS-R1 expression in iWAT was significantly decreased (p = 0.0041) compared to a LFD (Figure 6a). A 6 w HFD did not increase ghrelin receptor expression in rats compared to a 6 w LFD (Figure 6b). Following a 4-week exercise intervention, GHS-R1 expression was significantly increased in iWAT compared to LFD-fed (p = 0.0101) and HFD-fed (p = 0.0315) rats (Figure 6b), and RP AT (Figure 6b) compared to LFD-fed rats (p = 0.0041). There were no significant differences in CRFR-2 expression in iWAT or RP (Figure 6b).

## Discussion

### Overall findings

Ghrelin, a hormone that spikes before mealtime and decreases immediately after, has previously been shown to directly blunt beta-stimulated lipolysis in WAT, likely as a mechanism to decrease fatty acid mobilization prior to the consumption of meals. As little as 3–7 days of HFD feeding can impair substrate handling in WAT [15] 16]; therefore, we hypothesized that a short-term 5d HFD would impair the regulation of WAT lipolysis by ghrelin. To investigate the long-term effects of HFD consumption on ghrelin, we hypothesized that impaired ghrelin regulation of lipolysis could be restored with an exercise intervention. In the current study, the ability of ghrelin to blunt CL-stimulated lipolysis in iWAT *ex vivo* was lost with 5d of a HFD compared to a LFD as hypothesized. However, ghrelin’s ability to modulate lipolysis was not due to changes in HSL phosphorylation. Additionally, exercise failed to restore the ability of ghrelin to regulate lipolysis in iWAT. Our findings suggest that ghrelin’s ability to modulate WAT lipolysis is rapidly lost with short-term HFD consumption. Following 6 weeks of a HFD (with or without exercise), ghrelin did not have any effects on regulating WAT lipolysis.

### Ex vivo model eliminates confounding variables

Studies investigating the role of ghrelin on WAT lipolysis show contradicting results across different models. *Ex vivo*, ghrelin has been shown to blunt β adrenoreceptor-stimulated lipolysis in adipocytes [9], and in rat subcutaneous and visceral WAT [5] via the suppression of HSL, a major rate-limiting enzyme controlling triglyceride hydrolysis. Interestingly, these same effects were not observed *in vivo* following ghrelin infusion [5] 7, 8], highlighting the difficulty in assessing ghrelin’s independent metabolic effects *in vivo* due to confounding factors. Specifically, ghrelin is a potent stimulator for growth hormone (GH) secretion [13], which may inhibit insulin release and thereby allow lipolysis to occur relatively unimpeded. Furthermore, GH has been shown to promote lipolysis in WAT directly by activating HSL. Therefore, it is difficult to untangle the effects of ghrelin in a more physiologically relevant *in vivo* model compared to our *ex vivo* work.

### Short-term HFD consumption impairs ghrelin’s ability to blunt glycerol release from iWAT

Both AG and UnAG have been shown to reduce β-adrenergic stimulated lipolysis *ex vivo* in isolated adipocytes [9], and in cultured subcutaneous and visceral WAT explants in previous work published by our lab [5]. Although the acylated form of ghrelin is traditionally considered to be the more metabolically active form, we have shown previously that the unacylated form of ghrelin plays an active role in blunting CL-stimulated glycerol release in RP via changes in HSL phosphorylation, but not ATGL, another lipolytic enzyme [5]. The present study demonstrated that AG had a greater effect on blunting CL-stimulated iWAT lipolysis *ex vivo* than UnAG (which also appeared to decrease lipolysis but did not reach significance), and ghrelin’s anti-lipolytic effect was lost with 5d of consuming a HFD. Despite changes in adipose tissue HSL phosphorylation status in a previous study [5], the present study did not show differences in HSL phosphorylation that could explain differences seen in glycerol and free fatty acid release with ghrelin treatment. In accordance with our data, the protein content of the ghrelin receptor growth hormone secretagogue receptor type 1a GHS-R1a was decreased in iWAT of HFD-fed compared to LFD-fed rats. Ghrelin receptors (GHS-R1a) have been measured in pancreatic islet cells [17], and both isoforms of ghrelin play a role in the regulation of pancreatic β-cell insulin release [18], which has a strong effect on suppressing WAT lipolysis [19]. It is plausible that ghrelin also acts via receptors in WAT to directly modulate lipolysis. A recent study demonstrated that adipose tissue-specific deletion of GHS-R protected against HFD-induced insulin resistance as well as inflammation and fibrosis in adipose tissue, further supporting the hypothesis that ghrelin exerts its effects in adipose tissue via GHS-R [20]. To our knowledge, this study is the first to investigate the direct response of WAT lipolysis to ghrelin treatment in rats fed a HFD. Previous work has focused only on ghrelin secretion after high-fat meal consumption in humans, showing that consuming 1 or 2 high-fat meals may [21] or may not [22] impair ghrelin’s appetite regulating effects.

Unlike previous work in our lab using adipose tissue [5], ghrelin treatment did not blunt β-adrenergic stimulated lipolysis in the RP AT depot. However, this previous study used a standard chow diet (24% kcal protein, 18% kcal fat, 58% kcal from carbohydrate), while rats used in the current study were fed a LFD with approximately 7% kcal coming from added sucrose (20% kcal protein, 10% kcal fat, 70% kcal carbohydrate). It is not yet known how dietary macronutrient composition, such as the addition of sucrose, can affect ghrelin regulation of metabolism.

### Ghrelin’s ability to blunt iWAT glycerol release is lost with age/size, regardless of diet

Although we initially hypothesized that consumption of a HFD (short or long term) would impair ghrelin’s ability to blunt β-adrenergic stimulated lipolysis in WAT, we were surprised to observe that ghrelin’s anti-lipolytic effects in iWAT in rats fed a LFD for 6 w was also lost. However, the rats in the 6 w LFD group weigh more and were older than the rats in the 5d LFD group. Changes in the role of ghrelin with increased age has not yet been established and in the present study we simply cannot disentangle the effects of age and body/tissue weight. Ghrelin knockout mice have demonstrated resistance to ageing-related increases in fat mass and weight gain [23]. In humans, plasma levels of acylated ghrelin have been shown to decline with age even in healthy adults [24], although this past study did not investigate potential changes in ghrelin function. It is plausible that a gradual decline of ghrelin levels with healthy weight gain or age may result in the loss of some of ghrelin’s functions, including the ability to modulate WAT lipolysis. Future studies could explore the timing at which the regulatory effects of ghrelin on lipolysis are lost.

### Exercise does not restore ghrelin’s ability to blunt iWAT glycerol release

Results of studies exploring the effects of exercise training on basal and stimulated lipolysis in adipocytes isolated from humans are equivocal, as lower, no change, or higher basal lipolysis have been reported following exercise intervention compared to sedentary controls [12] 25, 26, 27, 28]. Whether ghrelin plays a role in potential exercise-stimulated changes in lipolysis is unknown. Some studies show an association between long-term exercise training and increased ghrelin levels when there is weight loss in human subjects [29] and rats [30], and no changes in ghrelin levels in normal-weight subjects [31], suggesting that regulation of ghrelin levels is dependent more on body weight than exercise. In a study by Mifune et al [30], HFD-fed rats had decreased serum ghrelin compared to chow-fed rats, which was restored to normal levels with voluntary wheel running; however, the study was not mechanistic in nature and did not explore ghrelin regulation of WAT metabolism. In our study, a 4-week exercise training intervention increased adipose tissue GHSR-1a content compared to the 6 w HFD-fed group with no exercise. However, regardless of this increase in ghrelin receptor content, we found no evidence that exercise training restored ghrelin’s antilipolytic effect in WAT. It is well established that exercise protects muscle from a HFD-induced loss of response to hormones like insulin and leptin [12]. Exercise acutely induces a stress response in activated muscle, such as increasing reactive oxygen species (ROS) production [32], triggering pathways that results in numerous physiological adaptations including increased mitochondrial biogenesis [33] and reduced inflammatory cytokine production [34]. Such effects may potentially protect or restore the response of muscle to various hormones. The results of the current study demonstrate that although the exercise intervention group had lower WAT depot weights, exercise was not able to restore ghrelin’s ability to blunt lipolysis in this tissue.

### Limitations

There are a few factors that could account for discrepancies between previously reported results. Since rats are fasted overnight and given food in the morning prior to the experiment, we are not certain when rats last ate. This could lead to variability in circulating ghrelin levels, and potentially affect how WAT responds to ghrelin exposure *ex vivo*. There are also regional differences in β-adrenergic receptor distribution between, and even within, WAT depots depending on the state of adipocyte differentiation [35] 36], as well as differences in lipolytic activity between depots [37].

### Conclusions

Acylated ghrelin blunts CL-induced lipolysis, measured by WAT glycerol release, in iWAT from young, small rats fed a LFD for 5d. However, both short-term HFD consumption and time (6 w, regardless of dietary fat intake) impaired ghrelin’s ability to blunt lipolysis. Exercise training did not restore the ability of ghrelin to modulate lipolysis. In this study, the observed reduction in lipolysis was not associated with altered phosphorylation of inhibitory or activating sites of HSL. These findings are the first to demonstrate the development of ghrelin resistance in WAT, and furthermore, that this cannot be rescued by chronic exercise. Future studies are required to elucidate in greater detail the signalling mechanisms by which ghrelin modulates adipocyte lipolysis, and particularly in the context of different lifestyle interventions like diet or exercise.

## Materials and methods

### Animal housing, experimental diets and tissue collection

Animal experimental procedures were approved by the Animal Care Committee at the University of Guelph (Animal Utilization Protocol #4121). Male Sprague-Dawley rats (6–7 weeks of age, weighing 150–175 g for 5-day experiments; 4–5 weeks of age, weighing 75–100 g for 6-week experiments) were obtained from Charles Rivers Laboratories. Rats were housed at 22–24°C in groups of 3–4 in cages with a 12-h:12-h dark: light cycle and fed *ad libitum* for either 5 days (5d) or 6 weeks (6 w) a low-fat diet (LFD; 10% kcal from fat; D12450J, Research Diets) or high-fat diet (HFD; 60% kcal from fat; D12492, Research Diets). Composition of diets is indicated in Table 1. Body weight and food intake were measured weekly. After 5d or 6 w of experimental diet, rats were anesthetized by intraperitoneal injection of 6 mg sodium pentobarbital/100 g body weight prior to collection of subcutaneous inguinal white adipose tissue (iWAT) and visceral retroperitoneal adipose tissue (RP).

**Table 1.** Composition of experimental diets. composition of experimental diets, provided by the manufacturer research diets inc., new brunswick, NJ, USA)

Formulation			
Class Description	Ingredients	D1245-J Rodent diet with 10% kcal fat	D12492 Rodent diet with 60% kcal fat
		Grams (g)	Grams (g)
Protein	Casein, Lactic, 30 Mesh	200.00	200.00
	Cystine, L	3.00	3.00
Carbohydrate	Starch, Corn	506.20	-
	Lodes 10	-	125.00
	Sucrose, Fine Granulated	72.80	72.80
Fiber	Solka Floc, FCC200	50.00	50.00
Fat	Soybean Oil, USP	25.00	25.00
	Lard	20.00	245.00
Mineral	S10026B	50.00	50.00
Vitamin	Choline Bitartrate	2.00	2.00
	V10001 C	1.00	1.00
Dye	Dye, Yellow FD&C #5, Alum. Lake 35–42%	0.04	-
	Dye, Blue FD&C #1, Alum. Lake 35–42%	0.01	0.05
Total		1055.05	773.85
Caloric Information: Physiological Fuel Values (% kcal)			
Protein		20	20
Fat		10	60
Carbohydrate		70	20
Energy Density		3.82 kcal/g	5.21 kcal/g

**Table 2.** Body weight, caloric intake, iWAT and RP depot weights in rats fed either a low-fat (LFD) or high-fat diet (HFD) for 5 days (5d). Bonferroni post hoc testing showed a significant increase (p < 0.05) in caloric intake, and iWAT and RP depot weight in HFD-fed compared to LFD-fed rats. All data are presented as mean ± S.E.M. (n = 11–12 rats/groups)

	LFD	HFD	p-value
Initial body weight (g)	151.9 ± 2.8	151.2 ± 1.9	p = 0.9969
Final body weight (g)	229.7 ± 3.2	239.0 ± 4.0	p = 0.1031
Total caloric intake (kcal)	324.4 ± 1.3	439.6 ± 11.8	p < 0.0001
iWAT depot weight (g)	2.3 ± 0.2	3.15 ± 0.40	p = 0.0305
RP depot weight (g)	1.0 ± 0.1	1.42 ± 0.15	p = 0.0110

### Exercise protocol

A subset of rats receiving a HFD for 6 weeks began an exercise training protocol after 2 weeks of HFD consumption. The exercise protocol continued for the remaining 4 weeks of the HFD. Rats were acclimatized to the treadmill prior to the start of the protocol by spending 5, 10 and 15 minutes on a treadmill. The protocol increased in intensity every week. In week 1, rats ran at a speed of 10 m/min for 1 hour at a 0% incline. For week 2, rats trained at a speed of 15 m/min at a 5% incline for 1 hour; in week 3, the speed was maintained at 15 m/min for 1 hour at a 10% incline, interspersed with 5 maximum effort intermittent sprints/day. In week 4, rats ran at a speed of 20 m/min for 1 hour at a 10% incline with 5 intermittent maximum effort sprints/day. This is similar to previous training protocols used in our lab [38] 39].

### Glucose tolerance test protocol in 6 wk-fed rats

During the 5^th^ week of diet, an IPGTT was performed on overnight fasted rats. Fasting blood glucose (t = 0 min) was measured via tail vein using a FreeStyle Lite glucometer before rats were injected with 2.0 g/kg body weight glucose solution. Subsequent blood glucose measurements were taken at t = 15, 30, 45, 60, and 120 min following injection.

### Ex vivo lipolysis and signalling

Both iWAT and RP (~350 mg) were collected from rats under anaesthesia for ATOC experiments. Tissue samples were minced and placed into warmed (37°C) and gassed (95% O_2_, 5% CO_2_) M199 media supplemented with 5 mM glucose, 1% antibiotic/antimycotic, 50 uU insulin and 2.5 nM dexamethasone. Some rats, particularly in the short-term diet portion of the study, did not have enough WAT for all ATOC treatments. In these instances, WAT samples from 2 rats were pooled to have sufficient quantities available for all treatments. After 24 h of incubation, media was removed and fresh media added to the ATOC samples. During this second 24 h incubation, the following treatments were added to the samples: control (CON), lipolytic stimulus (1 μM CL316,243; CL), lipolytic stimulus plus acylated ghrelin (1 μM CL+150 ng/ml AG), lipolytic stimulus plus unacylated ghrelin (1 μM CL+150 ng/ml UnAG). Concentrations of CL and AG/UnAG were identical to that previously used in our lab [5]. While the ghrelin concentration is higher than normal physiological levels, it elicits detectable effects in tissue based on previous dose-response work [9]. After 2 h of treatment, sampled media and WAT were snap frozen in liquid nitrogen and stored at −80°C until analysis.

***Glycerol quantification***As an index of lipolysis, glycerol content of the ATOC media was quantified using a sensitive glycerol assay kit (cat. nos. G7793, F6428, Sigma Aldrich; mean %CV = 7.35).

***Non-esterified fatty acid (NEFA) quantification***NEFA content in ATOC media was quantified using a free fatty acid assay (comprised of the following components: cat. nos. 995–34,791, 993–35,191, 999–34,691, 991–34,891, 276–76,491, Wako Diagnostics; mean %CV = 3.3). In conjunction with the glycerol measurement, this was used to calculate primary fatty acid reesterification in WAT as follows: (3 × glycerol release into media) – measured fatty acid release [40].

### Protein quantification and western blot analysis

WAT samples were homogenized in cell lysis buffer supplemented with phenylmethylsulfonyl fluoride (Roche) and protease inhibitor cocktail (Sigma Aldrich). Soleus muscle samples were collected and homogenized to analyse COXIV protein content to confirm a positive training effect. Samples were homogenized for 2 minutes and centrifuged at 4°C at 1500 rcf. Supernatant was collected and protein concentration was determined using bicinchoninic acid (Thermo Scientific). Protein samples were prepared with Laemmli sample buffer (Bio-Rad Laboratories, Inc.) and β-mercaptoethanol (Sigma Aldrich) to achieve a final concentration of 1 ug/ul. Protein was loaded and separated using a 10% SDS-PAGE gel, then transferred to a nitrocellulose membrane (Bio-Rad Laboratories Inc.) and blocked in 5% non-fat milk in Tris-buffered saline with Tween (TBST) for 1 h. Membranes were incubated at 4°C overnight in primary antibodies (1:1000 dilution): total HSL (cat. no. 4107, Cell Signalling Technology), phospho-HSL Ser 660 (cat. no. PA5-64,494, Invitrogen), phospho-HSL Ser 565 (cat. no 4137, Cell Signalling Technology), CRFR-2 (cat. no. ab104368, Abcam), GHS-R1 (cat. no. sc-374,515, Cell Signalling Technology), and COXIV (cat. no. ab16056, Abcam). Membranes were washed with TBST and incubated in either goat anti-mouse or anti-rabbit horseradish-peroxidase-conjugated secondary antibody (1:2000 dilution) in 5% non-fat milk in TBST for 2 h. Protein bands were imaged using ECL (Perkin Elmer) and Alpha Innotech software. All protein content was normalized to Ponceau S staining.

### Statistics

Statistical analyses were conducted using GraphPad Prism 8 software (GraphPad Software Incorporated). Body weight, weekly food intake and IPGTT data were analysed using a repeated measures two-way ANOVA test followed by a Bonferroni post-hoc test. IPGTT AUC was quantified by calculating peak area above baseline blood glucose concentration (mmol/L) at t = 0 mins for each animal. Adipose tissue depot weights and total food intake were analysed using a student’s t-test. A repeated-measures, non-parametric one-way ANOVA was performed for glycerol, free fatty acid, fatty acid re-esterification and Western blot data. All values are reported as mean ± standard error mean. A p < 0.05 was considered statistically significant for all analyses.
